# Methods to Characterize Effective Thermal Conductivity, Diffusivity and Thermal Response in Different Classes of Composite Phase Change Materials

**DOI:** 10.3390/ma12162552

**Published:** 2019-08-10

**Authors:** Elisabetta Gariboldi, Luigi P. M. Colombo, Davide Fagiani, Ziwei Li

**Affiliations:** 1 Dipartimento di Meccanica, Politecnico di Milano, Via La Masa 1, 20156 Milan, Italy; 2 Dipartimento di Energia, Politecnico di Milano, Via Lambruschini 4, 20156 Milan, Italy

**Keywords:** composite phase change materials, experimental methods, effective thermal conductivity, effective thermal diffusivity, heat flux probes

## Abstract

The phase change materials (PCMs) used in devices for thermal energy storage (TES) and management are often characterized by low thermal conductivity, a limit for their applicability. Composite PCMs (C-PCM), which combine active phase (proper PCM) with a passive phase with high conductivity and melting temperature have thus been proposed. The paper deals with the effect of length-scale on thermal characterization methods of C-PCM. The first part of the work includes a review of techniques proposed in the scientific literature. Up to now, special focus has been given to effective thermal conductivity and diffusivity at room or low temperature, at which both phases are solid. Conventional equipment has been used, neglecting length-scale effect in cases of coarse porous structures. An experimental set-up developed to characterize the thermal response of course porous C-PCMs also during active phase transition at high temperature is then presented. The setup, including high temperature-heat flux sensors and thermocouples to be located within samples, has been applied to evaluate the thermal response of some of the above C-PCMs. Experimental test results match Finite Elements (FE) simulations well, once a proper lattice model has been selected for the porous passive phase. FE simulations can then be used to estimate temperature difference between active and passive phase that prevents considering the C-PCM as a homogeneous material, to describe it by effective thermo-physical properties. In the engineering field, under these conditions, the design steps for TES systems design cannot be simplified by considering C-PCMs as homogeneous materials in FE codes.

## 1. Introduction

The phase change materials (PCM) used in devices for thermal energy storage and management are often characterized by low thermal conductivity [1,2], a limit for their applicability. A widely diffused class of PCM is that of latent heat PCMs, where thermal energy is stored/released by melting/solidification transition/s. Composite PCMs are another class of PCM, partly overlapped to Latent Heat-PCMs (LH-PCMs), defined as composite materials made of two phases: The active phase, which guarantees good latent thermal energy storage properties, and the passive phase, which must avoid phase changes during the entire range of working temperatures [1,3,4,5,6,7,8,9]. In the following paper, the focus will be on C-PCM in which the active phase is an LH-PCM and the passive phase has principally the thermal conductivity enhancement role.

**Figure 1:**
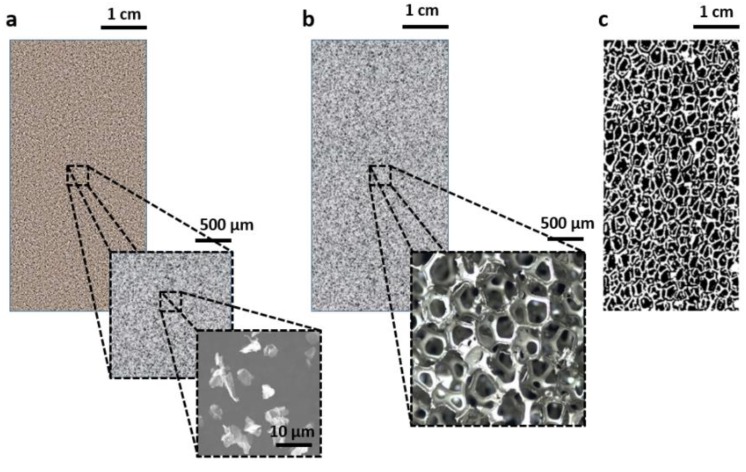
Representative length-scale of C-PCM materials related to the arrangement of the passive phases. Length scale of some tens of micrometer (**a**), of tenths of millimeter (**b**) and of some millimeter (**c**). Foam images in (b) and (c) adapted from [15], reprinted by permission from Springer Nature.

The selection of high-thermal conductivity passive phase, its suitable amount and distribution allows to overcome the abovementioned critical feature of PCM, and this is the most general reason for the production of C-PCMs. Further, in some cases, other structural or functional properties need to be associated with C-PCMs [3]. Examples of these C-PCM include the addition of graphene or graphite to polymeric PCMs [1,3,8,10], PCM impregnation into porous high-melting materials [1,3], micro-encapsulation [1,4], metallic alloys made by immiscible phases [5,6,7] can be adopted in PCM-based components. Considering high length scale C-PCMs, this can vary significantly from submicrometric to micrometric to hundreds of micrometers, to millimetric size (as shown in Figure 1). Porous materials, are characterized by the insertion of active phase into the pores (examples in Figure 1b,c). Due to the relatively regular arrangement of phases, they can be often considered as homogeneous materials at the length scale of some pore size, with specific thermophysical properties. Further, there is no clear distinction at a millimetric or bigger length scale between composite materials and composite structure, a term that is, in any case, mostly attributed to those, widely diffused, where high-conductivity passive phase materials are in the shape of circular and longitudinal finned tubes with various configurations, rings, and brushes inserted in cavities filled by PCM [1,11,12,13,14].

Several literature works have been devoted to the thermal characterization of homogeneous LH-PCMs and they are well summarized in reviews dealing with these materials [1,16,17,18] or specifically with their thermal characterization [19,20,21,22]. For LH-PCMs, the latter includes mainly evaluation of phase change temperatures and latent heat of melting/solidification (storage properties). As a matter of fact, the knowledge of these properties is fundamental in the choice of a PCM which satisfies both the temperature range application and the quantity of heat absorbed/released during the phase transition. They are obtained through experimental tests during which phase transition occurs and, for homogeneous PCM, these tests are based on isochronous differential scanning calorimetry [17].

On the other hand, thermal conductivity and/or thermal diffusivity, which can be considered heat transport properties and that during the stage of heat storage/release are related to the heat flow/removal from the region where phase transition is actually occurring, are usually determined below phase transition, when both phases are solid. The material class (metallic, polymeric, ceramics) of PCM and thus their typical melting temperature, conductivity/diffusivity properties, guide the selection of the test type [22]. In porous C-PCMs or structures their scale length should be taken into account as well.

Partly due to the novelty in considering C-PCMs composites as a specific material class and partly due to the relatively complex matter, to the authors’ knowledge, no specific standards for the thermal characterization of these materials are available. Only the review work by Zhang and co-authors on C-PCMs produced by combining porous passive phase with PCM [3] takes into account the thermal characterization of these materials. Specifically, the combination of two phases with significant differences in thermal conductivity, their arrangement and length scale drive the selection of thermal characterization techniques in C-PCMs. 

The present work gives an overview of different experimental methods proposed in the literature for C-PCMs and analyzes their applicability to characterize C-PCMs. Special focus is given to their applicability to the length scale of C-PCMs. An innovative experimental setup arranged to monitor the actual thermal response of C-PCMs with millimetric length scale porous structures will be illustrated and preliminary results will be provided. The results of the current version of the equipment will supply information on the actual thermal response of coarse porous C-PCMs during phase transitions. The novelty of the paper is that, combined with FE simulation, they will allow to find the temperature distributions in both active and passive phases under given boundary condition. This will lead to the identification of conditions under which the assumptions of homogeneous situations are met and for which conventional experimental tests can be adopted.

## 2. Methods

### 2.1. Tests to Measure the Thermal Storage Properties of C-PCMs 

**Figure 2:**
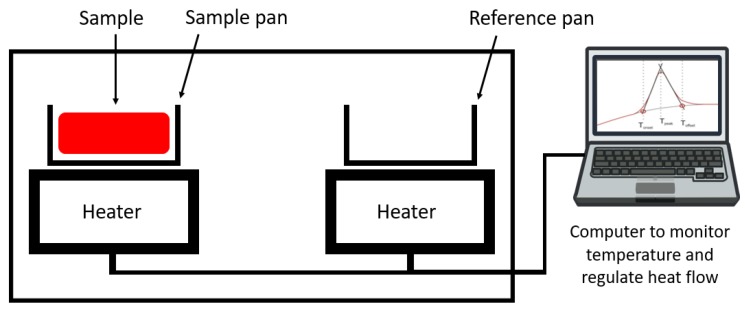
Schematic equipment for DSC analyses and typical DSC curve.

Phase change temperatures or temperature ranges and phase transformation enthalpies are the most important properties of PCMs. They are mainly characterized by differential scanning calorimetry (DSC) measurements [19,20,22], within other thermal analysis techniques [23]. In its classical version, a DSC apparatus includes two crucibles (or sample pans), one of which contains the sample, while the other is typically empty (see Figure 2). In classical tests of PCMs, when heating/cooling isochronous cycles, including phase transitions, are set to the crucible, the difference in the heat power supplied to the crucibles (or flowing from one to the other in some equipment arrangements) is related to the energy stored/released by the sample due to its phase transformation. Experimental data at constant heating/cooling rate are usually presented as DSC curves, heat flux/unit sample mass vs. temperature plots, as schematized on the computer screen in Figure 2, where phase transformations appear as peaks. From them, the temperature for the onset of transformation or the temperature range for phase transformation, and by integration, its enthalpy can be obtained. 

While the method is extensively used for homogeneous PCMs, in the case of C-PCMs it has some limitations related to the size of the crucibles. The latter have typically volumes of the order of some tens to a few hundreds of µL. In cases where the passive phase is finely distributed inside the PCM and the characteristic homogenization volume is equal or lower than the crucible one, DSC analyses can be properly performed on C-PCMs. Actually, a large number of papers analyzed DSC tests to detect changes in thermal energy storage properties as different mass fraction of finely dispersed passive phase used to enhance the thermal conductivity in the composite PCMs. Passive phases were expanded graphite (EG) [24,25,26,27,28,29], graphite matrix [30], nanosheets of graphene [31,32], graphene aerogel (GA) [33], sulfonated graphene (SG) [34], graphite nanoplates [35] and multi-walled carbon nanotubes (MWCNTs) [36,37].

DSC analyses were also performed on some porous C-PCM, in the cases of homogenization volume and size comparable to the crucible volume/sample size. This is the case of the work by Liang et al. [38], where graphene-nickel foam with pore size corresponding to case b in Figure 1 was filled with paraffin. Similarly, Xiao et al. [39] characterized C-PCM with paraffin as the active phase and the passive phase in the form of Al and Cu foams with 25, 10 and 5 pores per inches (PPI). The intermediate and coarser pores, roughly corresponding to case c in Figure 1, are relatively large for conventional DSC equipment/crucibles, even for some relatively big crucibles (about a hundred microliters or more) that are nowadays available. For relatively large porous C-PCMs or structures, thermal storage properties can be characterized by alternative measurement equipment and a method of analysis generally referred to as T-history method, revised in [23,40]. In these cases, equipment is based on the presence, in the same controlled-temperature environment (furnace), of a container with a reference sample without phase transitions in the investigated temperature range and of one/more containers for sample/s to be analyzed. Analyses of thermal response under similar conditions allow measurement of phase change enthalpy and specific heat of PCMs [23,40,41]. The volume of PCM samples can be far higher than that of DSC analyses and for several container shapes there is the possibility to test porous, encapsulated C-PCM, even if, to the author’s knowledge, there is no specific literature on it. Cases of T-history method application with controlled temperatures up to 120 °C have been reported [23], but due to the temperature range of interest for many organic PCMs and partly owing to the selection of water as a reference material, equipment versions, such as that proposed by Zhang et al. [40], cannot use T-history method for phase transitions close or above 100 °C. 

An alternative choice, more adopted for C-PCM structures than for porous C-PCMs, is to estimate the thermal energy storage properties on the basis of those characterized for their active phase: Transitions temperature are the same and enthalpy of C-PCM is derived from that measured for active phase once the volume fractions/densities of phases are known. Nevertheless, this method does not consider the possibility to have changes in the transition temperatures due to pressure variations arising from the volume constraint of the active phases in porous C-PCM, as demonstrated, for example, by Zhang [40].

### 2.2. Thermal Conductivity Measurements Methods

Thermal transport properties, such as thermal diffusivity, and conductivity, affect the time necessary to release/absorb heat by the material. Thermal conductivity is a thermal transport property used for the characterization both of massive PCMs and of C-PCMs. In this latter case, the main aim of the presence of a passive phase is actually an increase of the effective thermal conductivity, related to the presence and distribution of the passive phase. The addition of four mass% graphene nanosheets as the passive phase more than doubled the thermal conductivity of homogeneous PCM (from 0.34 to 0.91 W/mK) [31], while according to Mills e al. [30], the porous matrix of graphite filled with paraffin could lead to an increase the ETC of the C-PCM from 20 to 130 times that of homogeneous PCM, also with the possibility of obtaining anisotropic thermal conductivity related to phase arrangement of the passive phase. The beneficial effect on the acceleration of thermal response of a thermal energy storage system containing C-PCM is thus high. 

There is no one single technique that can be suitably used for measuring the thermal conductivity of all types of PCMs, as well described in [31,41]. They can be divided into two broad classes: Steady-state techniques and transient techniques and standard test methods have been proposed for many of them. Steady-state techniques, widely applied in the form of axial flow methods, require to reach steady-state conditions across the sample, which can take a long time and a rightful evaluation of the thermal resistance between the sample, sensors, and heating sources [42]. On the other hand, transient techniques work by measuring the temperature response to a constant heating power or a heat pulse supplied to the sample initially in thermal equilibrium and deriving effective thermal diffusivity from it. Among them, the hot wire method [43,44], the laser flash [45,46], and the transient plane heat source method [47] can be mentioned. 

The situation is far more complex when C-PCMs are taken into account. The effectiveness of a specific technique depends on different features, such as the measurement time, the required accuracy and reproducibility under various environmental conditions and, last but not least, by the physical and chemical properties of the material under investigation, its homogenization size and shape or “shapeability” of the C-PCM. Both steady-state and transient methods for ETC measurement and their applications to C-PCMs will be summarized in the following sections.

### 2.3. Steady-State Methods: Axial Flow Methods 

From a theoretical point of view, the thermal conductivity of a homogeneous material can be simply derived as the ratio between the one-dimensional heat flow per unit area in the sample (heat flux) and the thermal gradient along the sample, kept under steady-state conditions [42,48]. Different methods adopt different ways to keep steady-state conditions, to measure heat flow (or directly heat flux) and temperature gradient. Corresponding, different re-formulations of the above relation can be found in the literature for specific measurement techniques. Further, both cylindrical and prismatic samples are generally used. 

**Figure 3:**
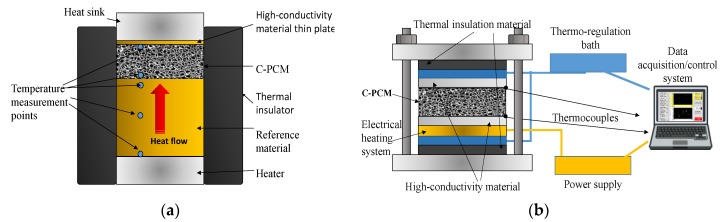
Axial flow methods: (**a**) A scheme of a comparative cut bar, (**b**) an example of the symmetric experimental setup (adapted after [11] with permission from Elsevier).

The most widely used method for axial thermal conductivity testing is the comparative cut bar (ASTM E1225 test method [42]), where the heat flow across the sample is derived from the thermal gradient along a reference sample of known thermal conductivity (Figure 3a). Often commonly, the sample is sandwiched between two reference samples (Figure 3b), to further account for minor heat losses that are very difficult to eliminate [3].

The comparative cut bar method was adopted in several studies to characterize C-PCM [30,31,49,50,51,52,53]. Different experimental setups adopted for C-PCMs are proposed in references [31,49,50,51,52,53], where reference samples were in different metallic materials, selected on the basis of the actual differences between their thermal conductivity and the sample ETC, of the maximum test temperature: Aluminum [49], copper [51], stainless steel [53] and indium [31]. A summary of the investigated C-PCMs, phases, and experimental parameters for axial flow methods applied to C-PCMs are given in Table 1. 

The largest temperature range derived from the examined papers hovers between 8 °C [30] and more than 200 °C [53], while the experimental set-up for unidirectional freezing developed by Feng et al. [52] has proved effective up to −7 °C. The thermal conductivity range derived from the examined papers lies between 0.3 W/mK [19] and 70 W/mK [49]. Sample sizes are actually dependent on the method and equipment adopted (see Table 1). The technique boasts high accuracy (uncertainty expected between 0.5 and 2.0%) [3] with respect to the other methods.

Under optimized conditions, and due to steady-state condition that allows several measurements, the ETC of C-PCMs can reach uncertainty between 0.5 and 2.0% [3], greater than in other methods and this is one of the reasons for their extensive use also for C-PCMs.

Nevertheless, some critical points and restrictions to its application directly derive from the need to meet the theoretical assumptions of the method, which are: Steady-state conditions, axial heat flow, and conductive heat transport only.

#### 2.3.1. Steady-State Conditions

The first assumption is directly correlated to the test duration (or at least to the time to be spent before reaching steady-state conditions), which is relatively long for materials of low ETC and high thermal inertia, within which some C-PCM.

#### 2.3.2. Axial Flow

The axial flow can be reached by keeping two opposite sample surfaces under temperature control and by minimizing lateral heat flow. For this reason, lateral thermal insulation with thermal conductivity far less than that of ETC of C-PCM sample has to be provided, or sample geometry should have a much lower thickness than the other sample dimensions, as exemplified by the setups schematically shown in Figure 3 [11,51]. Thermal losses also tend to increase with test temperature (to be kept over long times) and these tests, to the authors’ knowledge have not been performed above ~230 °C (500 K) [24]. The presence of axial flow also gives the possibility to measure the thermal conductivity of anisotropic C-PCM (for example, related to passive phase distribution, such as in Mills et al. [30], simply by testing samples with different passive phase orientations.

#### 2.3.3. Heat Transport Mechanism

Concerning heat transport mechanisms, both radiative and convective transports within the sample have to be minimized in axial flow methods. The first one has not been a problem for most of the tests carried out on C-PCM, since these were performed close to room temperature. The effect was considered negligible also in the case of C-PCMs tested just above 200 °C [24]. Convective heat transport has been accounted for in some studies on C-PCMs. The convection effect is known to affect the melting stage of structures containing homogeneous PCMs [50] while this heat transport mechanism generally slightly affects solidification. The confinement geometry of molten PCMs is among the factors affecting its convective motion in the molten state, to be taken into account in the design of experimental tests aimed at measuring the thermal conductivity of C-PCMs with passive phase distributed within the active phase. In the C-PCM with a porous passive phase, the length scale and shape of pores is mainly responsible for the presence and contribution of convective effects [50] to heat transport. Moreover, for C-PCMs the negligible effect of convection during solidification has been confirmed by Feng et al. [52].

#### 2.3.4. Thermal Gradient Measurements

Further requirements are to reduce experimental error measurements of thermal gradient. These can be obtained by increasing the heat flux, by increasing sample thickness (which reduces relative errors in temperature differences and in measurement points distances) or acting on both parameters. The second is preferred for materials with relatively high thermal conductivity, as can be the case for some C-PCMs. In the case of composite materials, an increase of specimen thickness should additionally be beneficial for the possibility to consider C-PCM as a homogeneous material. In case heat flow is increased, due to thermal contact resistance, the temperature difference between the sample and the surrounding parts conveying heat to/from the sample increases. TCR must be minimized and, when its effects are not negligible, it has to be taken into account in the estimation ETC. This is, for example, the case of most porous-C-PCM, where thermocouples are not embedded in the samples but stay close to it. TCR can, in these cases, be measured by means of either thermal measurements of samples’ of different thickness made of the same C-PCM [53] or of the same sample under different heat flows [31].

The beneficial role of applied pressure in minimizing TCR has been demonstrated for many materials [42,48]. In the case of porous C-PCM with relatively stiff Al foam, Sadeghi et al. [53] demonstrated that in the range of 0–2 MPa, TCR is relatively insensitive to compressive load in the range of 0–2 MPa. Similarly, for a C-PCM with copper foam passive phase, Feng et al. [52] demonstrated that natural contact, applied pressure but also bonding with a high thermal conductivity adhesive between the sample and the cold interface led to similar freezing rates for the investigated copper foam/water PCM system.

### 2.4. Transient Methods: Hot Wire Method

**Figure 4:**
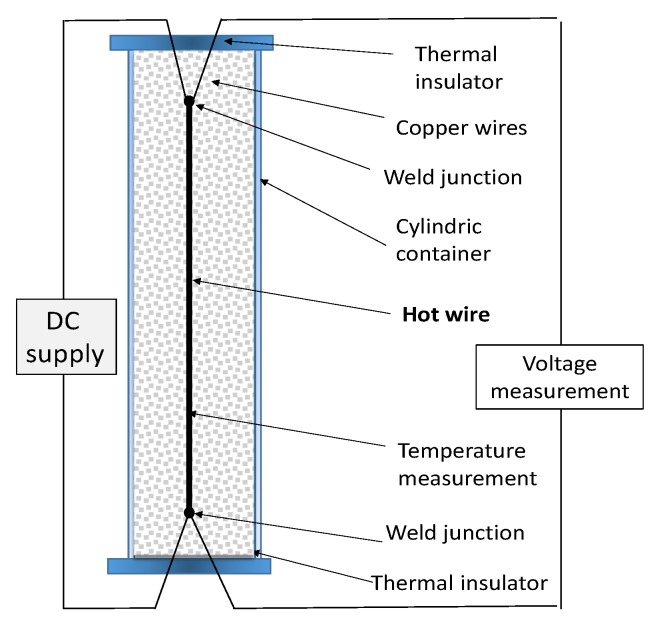
Schematic apparatus for hot wire tests (adapted from [56] with permission of Elsevier).

The hot wire method [43,44,55] is a standard transient dynamic technique based on the measurement of the temperature rise at a defined distance from a linear heat source (hot wire), a platinum wire embedded in the test material. If the heat source is assumed to have a heat output axisymmetric and uniform along the length of the test sample, which is kept constant from the beginning of the test at which sample temperature is homogeneous. A representative setup of the method is given in Figure 4, drawn after the work of Xiao [56].

The thermal conductivity arises from the analytical integration of the Fourier equation, which relates the temperature increase of the wire, power input, geometrical feature, and thermal conductivity, as given, for example, in [43,44]. By changing the initial temperature in different tests, the changes in thermal conductivity with temperature can also be derived. The activation temperature, sample size, and effective thermal conductivity values of the C-PCM whose effective thermal conductivities have been reported in the literature, as measured by this technique, are listed in Table 2. Some comments on the applicability of the hot wire method to C-PCM can be derived from these works. 

As far as the C-PCMs are concerned, those with finely dispersed passive phase particles are generally used. In these cases, the molten PCM can be poured in the container before testing. Semi-rigid C-PCM could also be characterized by this technique, especially in cases where wire and thermocouples are encapsulated in rigid, insulating parts [29]. 

As reported in literature and defined in standard practices [44,58], this measurement method should be applied only to low conductivity materials (0.02–15 W/mK), although Wang [36] and Hailot [57] have used it to successfully test samples with a maximum ETC of about 60 W/mK. The presence of the insulation layer could lead, in any case, to underestimation of thermal conductivity [55]. The application of the method to C-PCMs with porous passive phase is limited, more than the actual pore length size, by their rigid shape. In any case, the test can be applied in the case of isotropic materials. Concerning activation temperatures of C-PCM for which the method has been applied to C-PCMs, these are lower than 70 °C, often room temperature. Higher temperature could be critical and ASTM C115 upper limit is 1500 °C (taking into account radiative effects). In any case, an upper limit is represented by the temperature at which the material is no longer dielectric.

### 2.5. Transient Plane Heat Source (TPS) Method 

**Figure 5:**
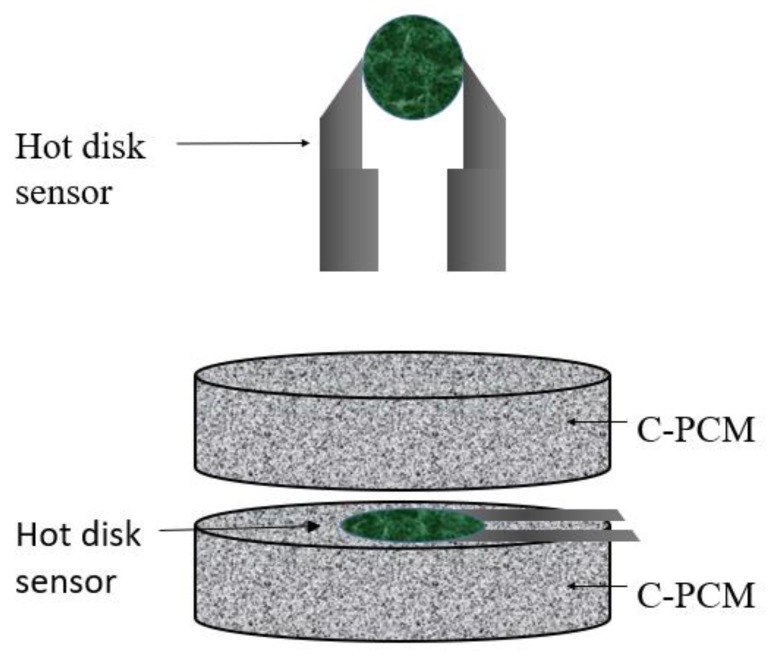
Schematic view of transient plane heat source method (adapted after [61] with permission form Elsevier).

The transient plane heat source (TPS) method is a transient technique, standardized in ASTM D 5930 [59]. TPS uses a thin strip or disk within which electric resistances act as a heat source [47,48,60,61]. The heat source shape results in alternative names given to the method of “hot plate” and “hot disk” tests, the latter being currently the most popular. Embedded in them or close to them are temperature sensors. The plate heat source is sandwiched between two plane samples of the material to be tested, as schematically shown in Figure 5. 

Starting from an initial condition of thermal equilibrium, a stepwise short heat flow pulse is supplied through the TPS. The increase in temperature at TPS is monitored. For a given heat flow, the temperature increase is proportional to the supplied heat flow pulse and inversely proportional to the thermal diffusivity of the sample [47]. Moreover, the temperature increase is directly proportional to the square root of the test time. Thus, thermal diffusivity can be derived once geometrical features, heat flow, and the evolution of the temperature at the sensor are known [47,48,59]. 

Similar to other transient methods, the plane hot source techniques allow quick measurements once thermal equilibrium of the system at the test temperature is reached (or a very slow temperature increase of the system is considered so that its temperature is almost homogeneous). Specific relations between these parameters and thermal conductivity are given in the literature referring to this method (see as examples [47,48,59])

The theoretical assumptions of the method, used in calculations to derive thermal conductivity, should also, in this case, be met in order to obtain good measurements. Among these, the ones to be carefully considered for C-PCMs are the usual assumption that only conductive heat transport occurs, with the same consequences discussed for previous test methods. 

Secondly, the theory has been developed for the case of symmetrical samples, infinite in length and width, with the possibility to measure together the thermal conductivity and thermal diffusivity of a homogeneous material. During the time of the measurement, the heat pulse has effects only within a distance that can be referred to as “penetration depth”, proportional to the square root of the material thermal diffusivity [59]. Only in cases where the distances of sample boundaries from the heat source exceed the penetration depth, the geometry of the sample has negligible effects on the measurement results [48,59]. Thus, both sample thickness and the lateral size exceeding the probe size should increase with material thermal diffusivity, which can be unknown at the beginning of the test and needs validation at the end of it. An additional requirement is that the penetration depth ranges roughly between 0.5 and 1 times the hot disk diameter [48]. The technique is suitable for a wide range of thermal diffusivity of samples, and thermal conductivity in the range from 0.005 to 500 W/mK has been measured [48], thus for a wide range of materials with different combinations of thermal conductivity, specific heat, and density, even if the abovementioned ASTM standard is for plastics. 

Another factor which can induce errors in the thermal properties derived by this method is thermal contact resistance, which alters the evolution of the thermal field within the system and thus can affect test results [47]. As in the case of axial flow methods, the specimen surface (as well as that of the probe) must be flat in order to minimize thermal contact resistance, as proved on C-PCMs [61]. Contact pressure should also be considered.

Among other theoretical features to be taken into account in view of a correct application of the method, the material needs to be homogeneous and isotropic [47]. Some modification of both technique and relations is needed in the case of orthotropic structures, provided that the heat flow direction is the axial one. In this case, test results are always related to thermal properties in both the axial direction and the transverse plane.

The above features have to be considered for the case of C-PCMs. 

Theoretically, the method could be applicable to C-PCM for which finely dispersed passive phase is embedded in the active phase, materials which have low thermal homogenization length compared to the typical sample thickness provided by standards for homogeneous materials of corresponding thermal properties. Thermal homogenization length might be thought of as the distance at which the thermal response becomes independent of the structure, and hence, it is a multiple of the characteristic length of the structure, i.e., five times or more.

Of course, these materials could be tested by plane heat source methods only below their activation temperature, as they can keep their shape. The survey on the scientific literature provided evidence of the application of this method to porous C-PCMs, tested at temperatures not exceeding 60 °C, as summarized in Table 3. The C-PCM had both inorganic (LiNO_3_/KCl eutectic [28]) and organic materials as active phases. In the latter case, paraffin [39,61,62], polyethylene glycol [26] n-octadecane [23] RT44HC [28]. As far as the passive phase is concerned, porous passive phases were often compressed expanded graphite, and the presence of thermal properties anisotropy has been considered in [29].

Xiao et al. [39] studied the passive phase in the form of metallic foams, with pore sizes ranging from 5 to 25 pores per inch (PPI). The pore structure with 5 PPI was relatively coarse with respect to the specimen thickness of 10 mm, and a relatively large experimental scatter was also observed in repeated tests.

### 2.6. Laser Flash Method 

**Figure 6:**
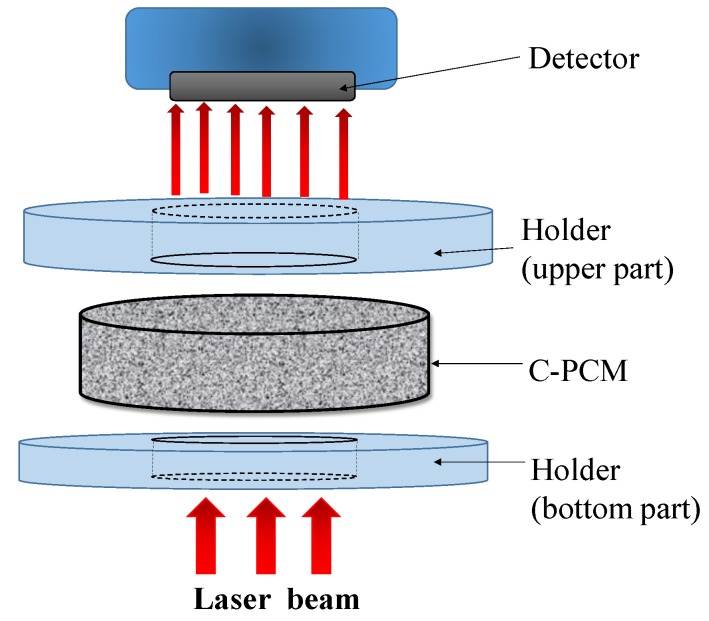
Scheme of the laser flash (LF) method applied to a C-PCM sample (adapted from [32] with permission of Elsevier).

The laser flash method is another method based on heat flow pulse experimental technique to measure thermal diffusivity [45,46,63,64]. The technique, schematically illustrated in Figure 6, derives its name from the use of a laser source to obtain a high energy pulse, whose duration is negligibly short compared to the time for its diffusion through sample thickness. During the pulse, the laser beam is homogeneously distributed within the sample, which is flat. Heat is absorbed by a sample surface blackened by graphite, which optimizes heat input also in the cases of transparent, semi-transparent, and reflective samples [63]. Heat is then considered to propagate axially along the thickness of a flat specimen. After the pulse, adiabatic conditions for the specimen are considered [63]. Once the sample, typically disk-shaped and initially to be in thermal equilibrium at the test temperature, is hit by the stepwise heat input flow on one side, its temperature change on the opposite sample side is recorded (Figure 6).

The above temperature increase can be theoretically modeled by considering axial conductive propagation of the heat pulse. A simple relation between the time t_0.5_ at which half of the temperature increase is reached, the sample thickness L and thermal diffusivity α is then derived [48]:

α = 1.388 × L^2^/t_0.5_, (1)

Thus, a simple analysis of experimental results leads to the calculation of the material thermal diffusivity [29,38]. As for the other methods, careful measurements can be obtained once experimental conditions reproduce at best the theoretical ones. In order to meet the assumption of axial flow and adiabatic conditions, the specimen contact with other parts of the systems is minimized, and non-contact (high-speed) temperature devices are generally used [45,46,63,64]. Adiabatic conductive conditions are also met by holding the sample in a vacuum environment (no convection transport in environment), by calibrating the temperature measurement device with a black body [45,63] and/or blackening both specimen surfaces by graphite [45]. Nevertheless, for very high-temperature measurements (exceeding about 1200 °C), corrections have to be considered during data analysis to take into account radiative losses [63]. 

The method is suitable for a wide range of temperatures and thermal diffusivity values (0.1–1000 mm^2^/s according to ASTM E1461 [46]), including high conductivity metals and thermal insulators, within which fall the thermal diffusivities of all C-PCM classes. 

Laser flash technique has been used for several C-PCMs, as summarized in Table 4. Notwithstanding the possibility to test materials at high temperature, mainly in the sample solid range, test data have been reported only up to 37 °C [34] for C-PCM, where active phases were all organic characterized by relatively low activation temperature. Sample discs were characterized by diameters between 10 and 20 mm, while sample thickness was in the order of 2–3.3 mm, conventional for LF technique, for which the small size of the specimen is one of the main advantages with respect to other measurement methods.

Among the assumptions for the applicability of the LF method [63], there is the homogeneity of sample material. This assumption also allows to derive the material thermal conductivity by multiplying the measured thermal diffusivity by the material-specific heat capacity at constant pressure and density. This has been done for C-PCMs for the case of passive phases of micrometric size, either dispersed into an active phase matrix or arranged to form a porous matrix to be filled by an active phase, such as, for example, in [32,33,34,65]. In these cases, specific heat and density of the composite have been either measured or calculated on the basis of those of the active/passive phases and their volume fractions in C-PCMs. The ETCs of the C-PCM listed in Table 4 range from 0.092 to 14.2 W/mK and confirm, in all cases, the enhancement produced by the presence of high-conductivity passive phase. As far as the investigated porous C-PCMs are concerned, the structure length size varied from some micrometers for the compacted graphite nanosheets tested by Chen et al. [32] to hundreds of micrometers (up to about 500) for porous Ni foam tested by Oya [66] and Liang [38]. In these studies the effective thermal conductivity of C-PCM was probably derived from thermal diffusivity as was done for the previously mentioned studies on C-PCM, independently of the representative length scale of the porous matrices and of the sample thickness and testing parameters (which are not always specified). 

The length scale of phases distribution in C-PCMs for LF measurements should be carefully considered. As discussed by Zhang et al. [3], for modeling the thermal response of C-PCM in transient situations, the different heat transport properties of the composite phases could generate non-equilibrium temperature conditions. In the case of LF tests, active and passive phase points laying at the same distance from the heat input surface could reach at a given time different temperatures. This is different from the theoretical basis for the derivation of the formulas used to derive thermal diffusivity by the LF method. A two-temperature energy equation related to active and passive phase, mainly focused to the situation of phase transition (for which also the contribution of natural convection within pores becomes important), has been developed. The model is quite complex to be applied to the case of the LF technique. Qualitatively speaking, it is clear that the classical assumption and thus the equation for homogeneous material in the LF method can be applied only in cases for which point-to-point temperature differences on the back surface of the sample are small, which is likely to occur if the sample thickness exceeds the thermal homogenization length, e.g., in the case of extremely fine length scale of pores or passive phase particles. 

Moreover, the effect of material anisotropy should be taken into account for some C-PCMs. The effect of passive phase orientation on the thermal transport properties has been demonstrated by Chen et al. [32]. The presence of axisymmetric properties and, at the same time, the difference in temperature which can be obtained in a composite structure at the temperature measurement surface has been considered by McMasters et al. [67] for a carbon fibers composite (not a C-PCM), in which case a careful elaboration of temperature data led to results not so different from those obtained by considering the material as homogeneous, even if with anisotropic properties.

It should be, in any case, considered that, beyond some tenths of a millimeter pore size, the measurement of thermal transport properties of composite material and the usual specimen thickness range, the laser flash apparatus becomes no more representative of the effective structure.

## 3. Experimental Setup for Thermal Response of coarse porous C-PCMs

Coarse porous C-PCM can be produced by filling with a PCM material (active phase) relatively large-sized (millimetric size) porous matrix of passive phase. When the former is an organic material and the second a metallic one, the thermal conductivity of the latter is two or three orders of magnitude larger than that of the active phase. The above C-PCM can be then used as a “PCM material” in larger thermal energy storage (TES) components, with a proper set of properties (within which ETC and ETD), which will simplify the design of the TES component and the evaluation of their overall thermal response, for example, by FE analyses.

Focusing the attention on the ECT, several models have been produced during the years to calculate it on the basis of the volume fractions of phases, in some cases also taking into account their arrangement [15,68,69,70,71,72,73]. Successful results were generally obtained. Among other factors, high TCR and contact surface between active and passive phases or by improper geometrical correspondence to a regular model might be considered, in some cases, as sources of discrepancies. The ETC of composite materials, (within which those of C-PCMs) have been modeled and typically tested in steady-state conditions, where no local temperature difference between active and passive phase exists. Nevertheless, as previously mentioned, discussing the applicability of transient methods to derive ETC of C-PCM, characterized by huge differences in thermophysical properties of the two phases, in transient conditions local temperature differences can be generated. These cannot be easily modeled, and, correspondingly, the ETC of the materials becomes dependent on the operating conditions [68,69] or the calculated ETC appears to be correlated to the pore size since as the pore size increases, the active/passive contact surface (per unit volume) reduces [73,74,75]. The effect of pore size also leads to the progressive importance of convection heat transport compared with conductive heat transport as, for the same active phase and temperature, pore size increases [74]. The combination of the two phenomena could lead to a pore size which optimizes, by making it faster, the thermal response of porous C-PCM in heating [74]. A complete understanding and modeling of thermophysical properties is of paramount importance in C-PCMs, whose applications are related to thermal cycles across the activation temperature of their active phase. Due to their assumptions, conventional methods cannot be used to consider what happens within a C-PCM during a thermal transient including phase transition. Steady-state methods, which actually can supply valid, effective, thermal conductivity also for coarse C-PCMs, cannot, by definition, be used during phase transitions. Transient methods measure the thermal diffusivity of homogeneous material, but in the case of a phase transformation occurring within the tested C-PCM, dealing with the test results to understand what is actually occurring within the C-PCM is not an easy task, even if the phase transition is simplified to occur at a single temperature.

There is thus the need to identify, by experimental tests and/or experimentally validated numerical models, what is the range of conditions where the material can be considered as homogeneous or to develop an experimentally validated model for effective thermal properties in transient conditions that it can be used to implement the C-PCM material properties in FE codes used to simulate the thermal or thermomechanical response of the overall structure. 

Since the above requests on experimental tests cannot be matched by the conventional transient techniques, an experimental setup design has been considered to perform experimental tests under different conditions on coarse porous C-PCMs. The considered test rig should potentially be able to test porous materials with pore size up to 7 mm and at temperatures up to about 300 °C. Of course, in addition to these porous-PCMs, other C-PCM or PCM structures with inhomogeneous structure could be tested. 

The experimental set-up was designed considering some previous examples of testing rigs to evaluate the transient thermal response of porous C-PCM with concrete matrix [54,74,75,76,77], using “modified” axial flow meter which allows dynamic conditions to be reached in the sample, considering either symmetric [76] or asymmetric [54,74,75] boundary conditions in the usual vertical heat flow [76,77] or in horizontal arrangement to check the effect of convection [74]. In the present case, the selection was for a system with a vertical heat flow arrangement. As a matter of fact, calculations performed for C-PCMs for which calculations of the Rayleigh number for the porous structures at the maximum temperature considered for the present work were lower the critical value for considering not negligible the effects of convection in porous structures heated from the bottom [77]. The possibility to monitor both temperature and the heat input/output at the two opposite surfaces was met by the use of heat flow probes, specifically the high-temperature FCR-200-M-K supplied by Wuntronic GmbH (Munich, Germany), with max service temperature 550 °C and max heat flux range 15,800 W/(m^2^K), sensitivity 560 (W/m^2^)/mV. Their circular flat surface is 15.9 mm diameter and made of stainless steel. They further acted as K-type thermocouples (measurement point E). 

**Figure 7:**
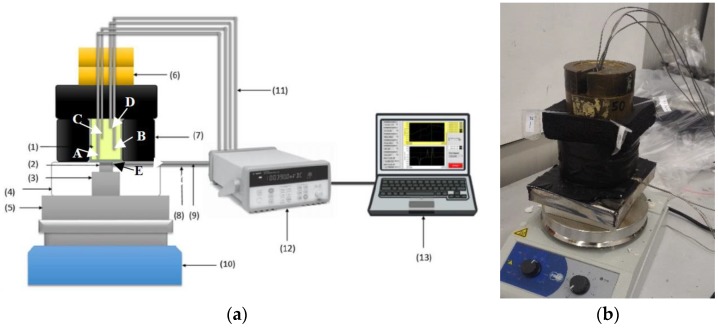
(**a**) Simplified representation of the “experimental set-up n. 4”: (**1**) Sample, (**2**) heat flux sensor, (**3**) Al cylinder, (**4**) base insulator, (**5**) Al plaque, (**6**) weight pans, **(7**) lateral insulator, (**8**) heat flux sensor’s cable, (**9**) K-type thermocouples at point E, (**10**) electrical hot-plate, (**11**) N-type thermocouples at points A–D, (**12**) mustimeter, (**13**) PC for data acquisition. (**b**) Picture of the experimental setup.

The system has been considered for C-PCMs with thermal conductivity higher than ceramic materials in [69] and the square cross-section samples were characterized by a length in the direction of heat flow twice as the lateral size, so that temperature measured at different positions along it will be relatively different. In the current development stage of the system, illustrated in Figure 7, a sample size of about 35 mm × 35 mm × 70 mm has been considered to test different course porous structures. The idea was to supply heat at one sample surface, while the other is thermally insulated, as well as the lateral surfaces. Further, samples of Al or Al alloys have been used the preliminary tests, with pore sizes ranging between about 10 and 5 PPI. In order to avoid leakage of molten PCM, 1 mm Al layer in thermal contact with the porous Al structure has been provided at specimen surfaces. Moreover, a 3 mm thick Al plate had been sandwiched between the sample and the heat flux sensor to homogenize temperature at the bottom of the C-PCM sample (point A). 

The transient response of the material (which can also be monitored by visual analyses in porous media with active phases becoming transparent as melting occurs [73,74]) was here more conventionally derived by means of thermocouples. Four Inconel-coated N-type thermocouples were placed within each sample, in PCM regions, at regular distances from the samples’ basis (A-0 mm, B-19.4 mm, C-40.4 mm, D-61.4 mm from the sample basis) and in symmetrical positions, along the cross-section diagonals at 12.4 mm from sample axis. Preliminarily, 2 mm diameter drilled holes allowed the correct positioning of thermocouple within each sample. In the current version of the system, neither temperature nor heat flow control is considered at the basis of the specimen. 

In order to monitor the inward thermal flux and to guarantee axial flux in the C-PCM, the heat flux sensor (16 mm in diameter) was connected via an Al cylinder through a homogenization 10 mm Al plate in contact with the heat source. Both the cylinder and the heat probe were embedded in alumina box to avoid lateral heat losses. On the other side of the probe, a 3 mm thick Al plate was placed to allow homogeneous temperature and axial flow at the bottom of the prismatic samples. 

The lateral and upper surfaces of the sample were thermally insulated with 32 mm thick K-FLEX ECO insulator, with thermal conductivity 0.040 W/mK at 25 °C and suitable for applications up to 150 °C. 

On the basis of the literature survey, particular care was taken to reduce TCR, specifically that between the interface of the Al plate and the sample and the probe. Nevertheless, since the temperature at the heat flux probe/plate was known (point E), the apparent thermal contact resistance between points A and E was derived as the ratio between these temperature difference and the heat flow. The (apparent) TCR was observed to be almost constant during tests, dependent on the actual positions of the sample, the Al plate below it and heat probe, and reduced by light pressure on the upper part of the thermal insulation system obtained by 20 N weight pans. 

## 4. Validation and Preliminary Tests

In the current version of the system, experimental tests in heating were carried out by setting the temperature of the bottom heater at a fixed value and simply monitoring both the heat flux and temperatures. The set of tests was carried out on specimens of the same geometry but characterized by different porous structures and active phase always of organic type. As the test setup was optimized during preliminary tests, TCR decreased from about 40 K/W to less than 10 and in the first set of tests actually carried out TCR ranged from 7 to about 20 K/W. 

**Figure 8:**
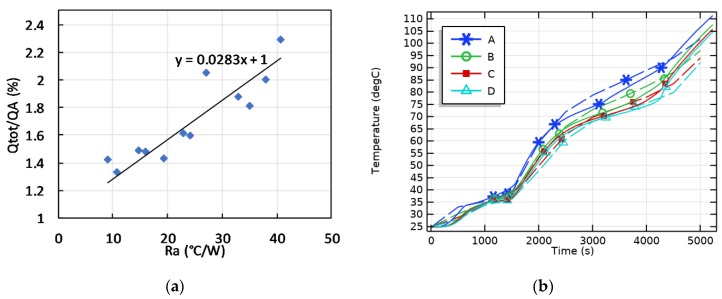
(**a**) Correlation between the thermal contact resistance (Ra) and the ratio between total heat entered at the bottom surface of the sample (Qtot) and that derived from probe heat flux (QA). (**b**) Comparison between experimental (solid lines) and modeled (dashed lines) temperatures at points A–D at different depth of the specimen, all laying in the active phase.

The preliminary as well as the first set of tests were used to validate the test set-up. The total heat entered in the sample at the time the minimum temperature reached 100 °C was calculated assuming the specimen were made of 4 overlapped parts, the temperature of which was the one read by the corresponding thermocouple. By knowing the phases volume fractions, specific heat, densities and the melting enthalpy of the active phase, considering as part of the sample also the Al box and considering adiabatic lateral and upper surfaces, the total heat entering the sample from its bottom surface was calculated (Qtot). It was observed that Qtot was always greater than the heat flow derived by integrating the heat flow passing through the probe in the same time span (QA). The analysis of all test data showed that as TCR increases, Qtot/QA increases (see Figure 8a), meaning that an increasing part of the total heat flow passes through the part of the Al system not in contact with the heat flux probe. The points derived from experimental tests have been fitted linearly, setting that the total heat flow is equal to that derived from measurements by the heat flow sensor in the case of zero TCR. The above correlation once more highlights the actual need to minimize TCR, as well as the need for validations of the system. The correlation between TCR and Qtot/QA represented by the fitting line in Figure 8 was considered to be valid at each test time and can be was used to derive the overall heat flow once known the heat flux at the probe and the temperature difference between it and the bottom thermocouple. A validation test was carried out by varying TCR by weight pan removal and repositioning during the test. This caused a sudden increase in thermal resistance from 7.5 to 33 and back to 7.5 K/W in an intermediate stage of the test. The result of these changes led to unusual temperatures-time curves (solid lines in Figure 8b). 

FE simulations were used to calculate, for the total heat flux history at the sample basis, the temperature evolution of the active phase in points A–D of a regular C-PCM structure modeling the porous passive phase. Results, shown in Figure 8b, are in very good agreement with experimental data, which validates the procedure adopted to calculate the total heat flow rate. The correspondence between experimental and modeled tests also suggest that the effects both of discontinuities in the passive phase and of local TCR between active/passive phases are negligible. Accordingly, FE simulations can be adapted to suitably design experimental tests in an improved experimental setup with temperature or heat flux control to derive ETC and ETD and their ranges of validity. 

The actual difference between total and measured heat flow prevents the possibility to compare the thermal history of different C-PCM materials under repeatable heat flow and temperature conditions unless in the case of close thermal resistance values. Nevertheless, the authors observed that, under several experimental test conditions, the thermal response of the C-PCM response was different from the expected one on the basis of the thermal response of a homogeneous PCM. The DSC curves of small size, homogeneous samples show well defined narrow peaks at the activating temperature. Vice-versa, constant heat flow leads to a sudden reduction of the heating rate of small-scale samples in the narrow temperature range at which phase change occurs. Greater mass sample can lead to broader peaks, which keeps a regular shape.

**Figure 9:**
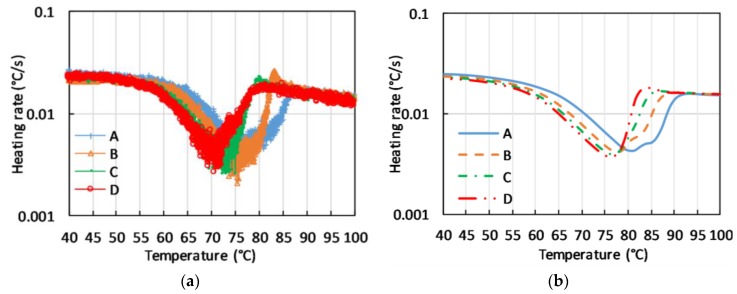
Experimental (**a**) and modeled (**b**) heating rate vs. temperature at points A–D, in the active phase of coarse porous C-PCM. FE model considered the porous material as a cubic rod-side lattice.

The thermal response of the macroscopic samples of C-PCM with coarse pores, presented in terms of heating rate vs. measured temperature at different positions of the sample, all located in the active phase region clearly display broad temperature ranges in which heating rate is reduced. Further, the presence of anomalous behavior, such as double peaks or sudden increases in temperature, can be noticed in Figure 9a. These effects are not only correlated to an extension of the temperature range for phase transition, but rather to the fact that at a fixed time the phase transition is at different stages of completion in different points of the sample. In these conditions, heat can be driven to or released by a part of the sample, and this can change the heating rate in other points of the sample. The phenomenon has also been described by FE simulations run for the same testing conditions (see Figure 9b). 

This abovementioned effect has been modeled by considering C-PCMs proving that the peak irregularities previously discussed, in addition to the active phase and its volume fraction, depended on the arrangement of the passive phase and on the heating range. Specifically, cubes lattice with square cavities as originally proposed by Dul’nev [72] have been considered to derive Figure 9b. The model had been previously successfully applied by Paek et al. to Al foams obtained by bubbling [15]. Other models based on the regular arrangement of unit cell structure have been proposed to model porous material or composite structure containing inclusions, such as body centered cubic and face centered cubic for foams obtained by bubbling processes [71] or the tetrakaidekahedral cell used for SiC foam [71], or even rod-side cubic structures for high porosity [78]. 

**Figure 10:**
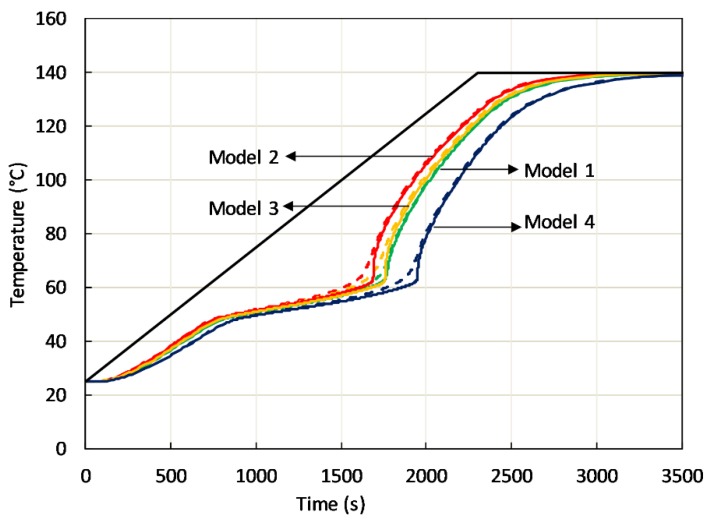
FE modeled temperature increase in two points laying at the same distance from the sample bottom in the active phase (solid line) and passive phase (dashed line), when sample bottom has a controlled temperature increase (black solid line). Each colour refers to a model considering a specific cell: 1: Rod-side, 2: Body centered cubic, 3: Primitive cubic, 4: Face centered cubic.

An example of the effect of passive phase modeling is depicted in Figure 10, where the predicted temperature evolution in two points at a distance of 17.5 mm from the sample bottom, one inside active phase, the other in the high-conductivity passive phase is shown. Among the previously mentioned models, rod-side (model 1), body centered cubic (model 2) and face centered cubic (model 4), also the simplest cubic cell containing spherical cavity (primitive cubic) has been considered (model 3). In all cases, isochronal heating at the sample bottom at 3 °C/min was considered. Figure 10 clearly shows that C-PCMs with the same effective specific heat and average density in the different arrangement display different heating in the temperature range where both phases are solid. It is also observed a different thermal response as far as the phase transition is concerned. Further, in all coarse C-PCMs, with modeled pore size of 10 PPI, a heating rate of 3 °C/min (0.05 °C/s) at the sample bottom leads to up to 10 °C temperature difference between close points in active/passive phase. It has been proved that temperature differences were actually reduced at reduced heating rate. 

The above simulations demonstrate that the actual arrangement of the passive phase can affect the temperature response of C-PCMs. Further, as previously mentioned, local TCR between active/passive phase could affect it in some cases. Thus, in general, FE simulations can be used to evaluate the range of operative conditions in which C-PCMs behave as homogeneous materials, but they need experimental validation under specific heating conditions. A more extensive, but carefully planned experimental test campaign is needed when the possibility to consider regular structures or to neglect local TRC are prevented. 

A wider range of experimental conditions could be met with the experimental set-up arranged to control heat flow or temperature at the bottom/upper surface, which is currently under development. 

## 5. Conclusions

The literature survey on steady-state and transient test methods used to derive thermal conductivity of C-PCMs was presented and the potentialities offered by each method to test coarse porous C-PCM were discussed. 

The current version of a complex experimental set-up aimed at characterizing the thermal behavior of coarse porous C-PCMs was presented, together with its validation and application under uncontrolled (but monitored) heat flow to C-PCM. 

Preliminary experimental results suggest heating rate changes occurring in an irregular way within a temperature range broader than that of phase transition derived from DSC tests on the active phase. FE analyses matched the experimental curves corresponding to points in the active phase. FE simulations on the modeled porous structures further demonstrated that for porous structures some millimeters coarse, 3 °C/min is a heating rate sufficient to lead to inhomogeneous temperature distributions among the phases during phase change, a situation preventing the possibility to consider the transient response of the C-PCMs as that of a homogeneous material. The results of FE simulations are partly related to the models used to represent porous structures, which should be selected carefully for each actual porous structure. 

The practical applicability of the experimental setup, coupled with FE simulations, consists of identifying the operational ranges within which the material can be considered to behave as a homogeneous material, making it easier the design of thermal energy storage systems and thermal energy management systems design. In the lab-scale field the method will provide information to check the applicability of transient methods for thermal conductivity characterization of PCMs. The experimental set-up here presented will be upgraded to allow the most complex and controlled boundary conditions needed to experimentally identify the abovementioned operational ranges.

## Figures and Tables

**Table 1:** Phases, experimental parameters and literature sources for axial flow methods on composite phase change materials (C-PCMs). In the table ND stands for ‘not defined’. TCR: Thermal Contact Resistance.

Composite PCMs	Testing T [°C]	k Range [W/mK]	Sample Size [mm^3^]	Insulation	Flux Meter Dimension [mm^3^]	Peculiar Aspects	Reference
CENG/paraffin CENG/hexadecane	ND	3–70	25 × 25 × 25	PS foam	25 × 25 × 65 (Aluminum)	Tested 2 commercial paraffin waxes	Py et al. [49]
Graphite matrix/paraffin	8/45	4–60	70 × 70 × 10	Unknown	70 × 70 × 17.55 (Stainless steel)	DSC analyses performed in different sample regions	Mills et al. [30]
Al foam/ paraffin	16/76	10–25	89 × 38 × 13	Silicon rubber	89 × 38 × 102 (Copper) 89 × 38 × 1.6 (Copper)	Determination of keff through the rule of mixture reveals to be erroneous.	Hong et al. [51]
NG/Unknown PCM ENG/Unknown PCM EGP/Unknown PCM	25/217	1–9	π(12.5)^2^ × 25	ND	ND	Graphite fins dispersion was optimized through mathematical simulations.	Pincemin et al. [24]
Al foam	ND	3–7	π(12.5)^2^ × 18	ND	π(12.5)^2^ × 45 (Iron)	The effect of TCR was evaluated.	Sadeghi et al. [53]
Graphene nanosheets/1-octadecano	ND	0.3–0.9	π(6.35)^2^ × 6.35	Teflon	π(6.35)^2^ × ND (Indium)	The effect of TCR was evaluated.	Yavari et al. [31]
Metal foam/Unknown PCM	+12/−7	1.95–3.92	30 × 40 × 50	Cotton (lateral) PMMA (top)	30 × 40 × 4 (Copper)	Tested different contact conditions; images taken to monitor cooling	Feng et al. [52]
Multi Walled Carbon NanoTubes/RT65 paraffin	10–40 °C	0.195–0.24	π(50)^2^ × 39	ND	ND	The effect of up to 1% MWCNT tested	Alshaer1 et al. [54]

**Table 2:** Phases, experimental parameters and literature sources for hot wire tests on C-PCMs.

Composite PCMs	Testing T [°C]	k Range [W/mK]	Sample Geometry [mm^3^]	Wire Insulation	Wire Sizes [mm]	Peculiar Features	Reference
EG/paraffin	10/70	0.4–0.8	32 × 22 × 105	ND	d = 0.18 l = 105 (Platinum)	The EG wt.% optimum was around 10.	Sari et al. [25]
MWCNT/ Paraffin	15/65	4–60	ND	Alumina	d = 0.07 l = ND (Platinum)	The mechanism of *k* enhancement caused by MWNT addition needs further investigation.	Wang et al. [36]
CENG/paraffin; CENG/ stearic acid; CENG/ pentaglycerin	25	5–60 (radial); 2–9 (axial)	25 × 25 × 25	ND	ND	The radial and axial thermal conductivity were distinguished due to anisotropy.	Haillot et al. [57]
EG/NaNO_3_; EG/KNO_3_; EG/ NaNO_3_-KNO_3_	ND	0.15–0.30	ND	ND	d = 0.03 l = 116 (Platinum)	Quadratic parallel model was successfully used to predict the *k_eff_*.	Xiao et al. [56]
MWCNT/stearic acid/PVP; EG/stearic acid/PVP; Graphene/stearic acid/PVP	ND	0.17–0.21	ND	Teflon	d = 0.05 l = 190 (Platinum)	The study also presented an experimental apparatus to evaluate the PCM performance during cooling.	Choi et al. [37]
Graphite nanoplates/ myristic acid	ND	0.15–0.21	ND	ND	d = 0.04 l = 19 (Nickel)	DSC analysis were performed to evaluate thermal reliability after 100 thermal cycles.	Ince et al. [35]

**Table 3:** Phases, experimental parameters and literature sources for transient plane heat source tests on C-PCMs.

Composite PCMs	Testing T [°C]	Thermal Conductivity [W/mK]	Sample Dimension [mm^3^]	Commercial Device	Reference
EG/polyethylene glycol	25	0.3–1.4	ND	TPS2500, Hot Disk Inc., Göteborg, Sweden	Wang et al. [26]
Cement mortar-EG/n-octadecane	25	1.8–2.2	100 × 100 × 10	TPS2500, Hot Disk Inc., Sweden	Zhang et al. [27]
Nickel foam/ paraffin Copper foam/ Paraffin	ND	0.3–4.9	100 × 100 × 10	TPS2500, Hot Disk Inc., Sweden	Xiao et al. [39]
EG/LiNO_3_-KCl	25	4–16	ND	TPS2500, Hot Disk Inc., Sweden	Huang et al. [28]
EG/paraffin	ND	0.7–14.0	π(30)^2^ × 30	TPS2500, Hot Disk Inc., Sweden	Li et al. [62]
LPE graphene/paraffin	7/42	0.1–85.0	ND	TPS2500, Hot Disk Inc., Sweden	Goli et al. [61]
EG/RT44HC	30/60	4–16	π(20)^2^ × 10	TPS2500, Hot Disk Inc., Sweden	Ling et al. [29]

**Table 4:** Phases, experimental parameters and literature sources for laser flash tests on C-PCMs.

Composite PCMs	Testing T [°C]	Thermal Conductivity [W/mK]	Sample Dimension [mm^3^]	Commercial Device	Reference
Carbon nanofibers/paraffin	25	0.25–3.00	π(9)^2^ × 3.3	ND	Elgafy et al. [65]
Porous nichel/erythriol	25	0.861–14.2	π(5)^2^ × 2	TC-7000, Rigaku, Tokyo, Japan	Oya et al. [66]
Graphite nanosheets/ paraffin	ND	0.33–4.47	ND	LFA-447 Nanoflash, NETZSCH, Selb, Germany	Chen et al. [32]
Graphene aerogel/ octadecanoic acid	25	0.184–2.635	π(6)^2^ × 2	LFA-447 Nanoflash, NETZSCH, Germany	Zhong et al. [33]
Sulfonated graphene/ polyethylene glycol	37	0.263–1.042	π(5)^2^ × 2	TC-7000H, Sinku-Riko Inc., kanagawa, Japan	Li et al. [34]
Polydimethylsiloxane-graphene-nickel foam/paraffin	25	0.092–1.626	ND	LFA-447 Nanoflash, NETZSCH, Germany	Liang et al. [38]
